# Transitional Cancer Care Program from Hospital to Home in the Health Care System of Iran

**DOI:** 10.31557/APJCP.2021.22.4.1231

**Published:** 2021-04

**Authors:** Zahra Alizadeh, Camelia Rohani, Maryam Rassouli, Mahnaz Ilkhani, Maryam Hazrati

**Affiliations:** 1 *Department of Medical-Surgical Nursing, School of Nursing and Midwifery, Shahid Beheshti University of Medical Sciences, Tehran, Iran. *; 2 *Department of Health Care Sciences, Palliative Research Center, Ersta Sköndal Bräcke University College, Campus Ersta, Stockholm, Sweden. *; 3 *Department of Community Health Nursing, School of Nursing and Midwifery, Shahid Beheshti University of Medical Sciences, Tehran, Iran. *; 4 *Cancer Research Center, Shahid Beheshti University of Medical Sciences, Tehran, Iran. *; 5 *Community Based Psychiatric Care Research Center, Shiraz University of Medical Sciences, Shiraz, Iran. *

**Keywords:** Cancer patient, transitional care, home care, integrated care

## Abstract

**Objectives:**

Transitional care program refers to the health care continuity during transferring from one health care setting to another or to home. This is an essential program for cancer patients and reduces the risk of unnecessary hospital admissions as well as the complications of the disease. The aim of this study was to develop a transitional cancer care program from hospital to home in the health care system of Iran.

**Methods:**

This study is a health policy and system research. It was conducted in four stages from October 2019 to January 2020. The first stage was a qualitative study. The qualitative data were collected through semi-structured interviews with 24 participants and a focus group with eight experts. In the second stage, a literature review of transitional care models was carried out. The initial version of the transitional cancer care program was developed based on the qualitative results and the literature review in the third stage. The validity and feasibility of the program were assessed using the Delphi study in the fourth stage.

**Results:**

Six major categories were extracted from the qualitative results, consisting of “integrated services for the continuity of care”, “holistic care”, “care standardization”, “the use of telemedicine”, “the transparency of rules” and “the care process provision”. Using these results and extracted the three common models of transitional care, the initial program was developed in three phases of pre-discharge, post-discharge, and transitional care with six protocols. The content validity of the program (98.7%) and its feasibility (95.8%) were approved by experts in the Delphi rounds.

**Conclusions:**

It is necessary to revise hospitals’ discharge program, and home health care center’s plan for admission and delivering health care services for cancer patients. Also, a pilot program is necessary to find the system advantages and disadvantages.

## Introduction

Care transition from hospital to home is an important strategy to continue care and to reduce the number of hospitalizations as well as the complications of the disease (Lima et al., 2018). It has been introduced by the World Health Organization as a solution to ensure health care services quality (Storm et al., 2014). Transitional care is a set of actions designed to transfer patients between different settings or different levels of care (Coleman and Boult, 2003). These settings include hospitals, sub-acute and post-acute nursing centers, patient’s homes, specialized and primary care centers, and long-term care centers (Hennessey and Suter, 2011). The effective and efficient management of transition from hospital care to primary care and vice versa is essential (National Transitions of Care Coalition, 2008).

In care transition, home care is an important part of patient care, the continuity of which is essential for the patient to achieve optimal health goals (Santomassino et al., 2012). Home care, as a community-based care approach, is one of the best ways to provide services to cancer patients and palliative care provision (Rassouli et al., 2017). Home care can also be an inseparable part of the post-hospital recovery procedure (transitional care), especially in the first weeks after discharge, when the patient still needs some level of regular physical assistance (WHO, 2015). This type of care can decrease care costs, increase patient satisfaction, reduce the length of hospitalization, and reduce hospital-acquired infections (Behm, 2015). The results of previous studies show that most developed countries have guidelines and models for the provision of these types of services in the country, which is designed based on their demographic, cultural and health care system structures (WHO, 2012).

However, in the present health care system of Iran, home care is on the rise as a novel care approach and is not yet sufficiently institutionalized in this structure (Heydari et al., 2016). Today, some hospitals affiliated to universities of medical sciences offer home care services to cancer patients using different approaches and available facilities (www.behdasht.gov.ir). Since each country should provide home care services for its people, according to the developed guidelines and in an organized manner, this study was conducted aiming to develop a program for cancer care transition from hospital to home in the health care system of Iran.

## Materials and Methods

*Design*

This study is a health policy and system research (Gilson et al., 2012) and part of a larger study. It is conducted from October 2019 to January 2020 with the ethical code of IR.SBMU.PHARMACY.REC.1397.096 from the Research Ethics Committee of Shahid Beheshti University of Medical Sciences. This study was designed in three stages, including a qualitative study, a literature review and a Delphi study. 

*Stage one*

*Design, Participants and Data Collection*

In the qualitative study, 24 participants were selected by purposive sampling from three major universities, a university hospital, Ministry of Health and a private cancer prevention and control center (MACSA) in Tehran. They consisted of three cancer patients, four family caregivers, 10 healthcare providers, three health policy makers and four faculty members of nursing schools. Inclusion criteria for selection of the healthcare providers (i.e. physicians, nurses, assistant nurses, head of home health care center, home health care services coordinator, home care experts and policy makers in the Ministry of Health and faculty members), were having experience and expertise in the cancer field and home care at least for five years.

The inclusion criteria for patients selection consisted of having a cancer diagnosis for at least one year and having been receiving home care services for at least 6 months. In addition, the family caregivers were selected who lived in a common place with the patient, and were directly involved in the home care of their patients.

In the qualitative stage, the data was collected through semi-structured in-depth interviews in a quiet environment. Individual interviews lasted between 20 and 60 minutes. The interviews began with a general open-ended question. A sample of the questions asked from patients is, “please tell me about your experiences of the care you received at home immediately after discharge from hospital”. A sample question for family caregivers is, “please explain about your experiences of using the services of home care centers”. A sample question for healthcare providers is, “please explain about your experiences of the current procedure of continuing care provision for cancer patients after discharge”. During the interview, questions such as, “can you explain more?” and “can you give an example?” were asked so that the participants would explain their experiences more deeply. All the interviews were conducted after the participants were informed of the research objectives and signed the informed consent form for the interviews and audio recording. In addition, all the participants were assured of the confidentiality of data and the right to voluntarily withdraw from the research at any stage.

In addition, a 90-minute online focus group with eight experts was held via sky room to enrich the results of the individual interviews and to enhance the rigor of the collected data. The participants were selected from the experts who participated in individual interviews with their informed consent.

*Trustworthiness*

The four criteria of Lincoln and Guba were examined, including credibility, dependability, conformability and transferability to ensure data accuracy and trustworthiness (Polit and Beck, 2017). Data validation was performed through prolonged engagement with data and spending time to collect and analyze data by the researcher. In order to increase the dependability of the data, the extracted codes were returned to some of the participants, to confirm the accuracy of codes. Moreover, data analysis was checked by five external reviewers, including three doctoral nursing students and two faculty members of nursing schools. To achieve conformability, all the stages of the research, especially data analysis, were recorded in detail. Through variation in the selection of participants from different places, the transferability of the data has been accomplished.

*Data Analysis*

The conventional content analysis based on Lundman and Graneheim’s approach was used to analyze the data (Graneheim and Lundman, 2004). All the interviews were recorded by a voice recorder, and immediately after each interview, the interviews were written down verbatim. The text of each interview was read and reviewed several times. Then, meaning units and primary codes were extracted and similar codes were placed in the same subcategory and similar subcategories formed categories. Data analysis was performed using MAXQDA version 10.

*Stage two*

In the second stage, a literature review was conducted by searching through the databases Web of Science, PubMed, Scopus and Google Scholar from January 1, 2000 to September 1, 2020 in order to obtain the most common and widely used models of care transition from hospital to home. The keywords “care transition”, “hospital discharge”, and “model” were used separately or alone with the keywords “home care” and “cancer” through Boolean expressions (AND, OR, NOT) by the first author. Inclusion criteria for selection of the articles included all the original articles in English which focused on the models of patient care transition from hospital to home. The first author and a specialist librarian, separately screened title, abstract and text of all the extracted articles.

*Stage three*

In the next step, based on the extracted models and qualitative results, an initial draft of the transitional cancer care program from hospital to home was created by the research team members.

*Stage four*

In the fourth stage, the cancer care transition program was assessed using comments made by 23 experts in two Delphi rounds, and the validity and feasibility of the program were evaluated. In this stage, experts consisted of three health policy makers, nine faculty members of nursing schools, eight nurses, two family caregivers and the head of a home care center. To evaluate expert opinions on the program, a 26-item researcher-made questionnaire with a 3-point Likert scale (high, moderate and low with the scores of 1 to 3) made up. Qualitative content validity of the questionnaire was examined by five nursing school faculty members. A score equal to or below 1.5 for each item was considered as disagreement. The range of agreement was a score of two or higher for each item. The data obtained by two Delphi rounds were analyzed using SPSS version 20 and the mean and the standard deviation of each item were calculated. 

## Results

*The result of the qualitative stage*

The mean age of the participants was 41.47 ± 8.98 years. Also, 45.9% of participants (n=11) was male and 54.1% was female (n=13). 

A total of 546 codes, six categories and 23 subcategories were extracted by analyzing individual interviews (Table 1). The categories included “integrated services for the continuity of care”, “holistic care”, “care standardization ”, “the use of telemedicine”, “the transparency of rules” and “the care process provision ”. A sample of participants’ quotations related to each subcategory was shown in Table 2 (Supplementary File).

*Category 1: Integrated services for the continuity of care*

Health care delivery systems are responsible for providing health services for individuals in the society and should consider the whole range of care from diagnosis and prevention to diagnostic, rehabilitation and palliative care as well as all the levels of service delivery in order to provide integrated health services for achieving universal health coverage. The category includes three subcategories (Table 1).

*Category 2: Holistic Care*

Holistic care is a comprehensive care approach provided by the home health care team in all physical, psychological, social and spiritual aspects of cancer patients, where multi-dimensional services are provided for patients. This type of care requires interpersonal and interdisciplinary cooperation within the health care team in order to improve the level of health and the quality of life (QoL). This category consists the two subcategories (Table 1). 

*Category 3: Care standardization*

Standard-based measures such as clinical packages and guidelines improve the quality of the services provided. These clinical packages can define how to provide services in different settings for different types of cancer, as well as insurance tariffs for different patients. Access to comprehensive care and guide like clinical package and guideline in different clinical situations, can be a good guide for health care providers to provide standard care. This category includes three subcategories (Table 1).

*Category 4: The Use of Telemedicine*

Telemedicine is the use of communication technology for clinical patient care. It includes four subcategories (Table 1).

*Category 5: The Transparency of Rules*

Due to the growth of long-term care services and the preference of patients and families towards home care, attention to the legal and ethical issues has become increasingly important. Therefore, making transparent rules can reduce abuse and other related problems. This category includes three subcategories (Table 1).

*Category 6: The Care Process Provision*

The purpose of providing cancer care is to improve the QoL by focusing on self-care and the empowerment of the patient and the family. This category consists of six subcategories (Table 1).

*The results of stage two*

After searching in databases, 80 studies were extracted. The titles and the abstracts of all extracted articles were studied separately by the first author and a specialist librarian. In the first screening step, 56 duplicate and 13 irrelevant studies were excluded and only studies, containing “transitional care models” were maintained. Finally, the full text of 11 articles were studied and three commonly developed models of patient care transition from hospital to home were extracted. They included Coleman’s care transition model, Naylor’s care transition model and community-based care transition model) Naylor, 2004; Coleman et al., 2006; Hennessey and Suter, 2011 ). 

*The results of stage three*

In this stage, after three sessions of discussion in the research team according to the qualitative results and the three developed models of care transition, the initial diagram of the process of transitional care program in cancer patients from hospital to home was developed in the three phases: pre-discharge, post-discharge, and transitional care with six protocols. 

*The results of stage four*

**Figure 1:**
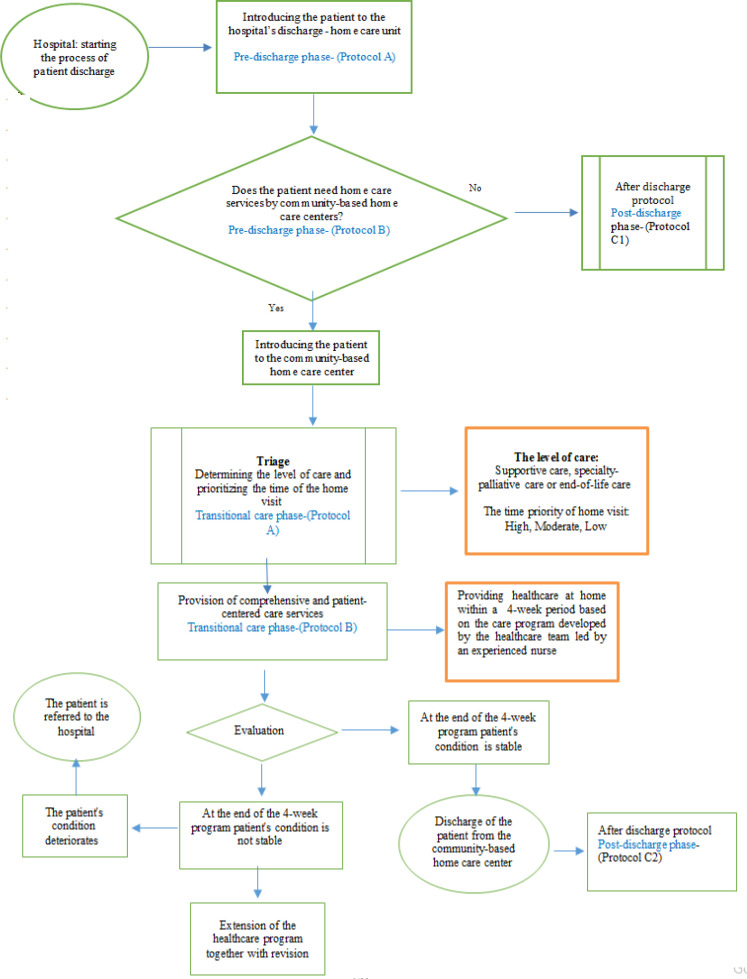
Diagram of the Transitional Cancer Care Program from Hospital to Home

Thirteen female (56.5%) and 10 male (43.5%) experts participated in the Delphi study. In the first round, 23 electronic files containing the diagram and protocols as well as a questionnaire for evaluation of the program, were sent to the experts by email. At the end of the first round, only 18 completed questionnaires were returned by email. Based on the scoring of the questionnaire, the items with mean score ≤ 1.5 were excluded and those with mean scores of 2 and above were accepted and entered the second round. In the second round, the modified program from the first round and the same evaluation questionnaire was submitted to the 18 experts who had completed the first phase by email. Only 16 experts completed the questionnaire and returned it by email. All items had a mean score ≥ 2. The results showed that the validity (98.7%) and feasibility of the program (95.8%) with three phases (Figure 1) and 6 protocols (Supplementary File) was satisfactory.

The program starts from the hospital’s discharge-home care unit when a follow-up nurse visits the patient and family. If the patient needs transitional care, he/she is entered to the transitional care phase, otherwise continues post-discharge phase and follow-ups program (Figure 1). 

**Table 1:** The Results of the Qualitative Study Including Categories and Subcategories

Category	Subcategory
Integrated services for the continuity of care	Universal access of clients to health services Integrated palliative care at different prevention levels Development of guidelines for health care transferring to home
Holistic care	Providing spirituality care, in addition to routine cares Interpersonal and interdisciplinary collaboration team
Care standardization	Developing clinical guidelines for home care Developing packages for home health care services Periodic monitoring
The use of telemedicine	Establishing an electronic platform of care transition system Registering patients’ electronic medical records on the system Accessing the care transition staff to the electronic system Establishing a telephone consultation system
Transparency of rules	Establishing a legal framework for home care Rules for entering the patient’s house Rules in case of errors
Care provision process	Involving the patient and family in the care process Assessment of the patient and family Identifying the existing and the potential problems of the patient and family Planning based on the abilities of the family for caring Evaluating the care program along with the patient and family Training the patient and family with focus on the self-care and empowerment

## Discussion

The purpose of this study was to develop a care transition program from hospital to home for cancer patients in the health care system of Iran. A cancer care transition program in three phases (pre-discharge, post-discharge, and transitional care) with six protocols were produced. Providing health care services from hospital to home is called hospital-based home health care services (WHO, 2004). 

Based on the needs of our cancer patients and their family, we started our program from the time of the patient’s hospital discharge, consistent with other studies (Naylor, 2004; Coleman et al., 2006; Hennessey and Suter, 2011; Heydari et al., 2016). Prior to discharge from the hospital, a primary assessment should be done and determine whether the patient needs to be referred to home health care centers or not, and how much is the ability of the patient and his/her family for self-care. Earlier studies showed that for improving patients and their families QoL, during the primary assessment the needs of patients and their families for home care services should be identified (Hennessey and Suter, 2011). 

In our study, planning for discharge phase was inspired by two models; Coleman’s care transition model (Coleman et al., 2006) and the community-based care model (Hennessey and Suter, 2011). At this phase, the nurse interviews with the patient and his/her family and makes the needs and problems list. Weiss et al., (2015) explained that the discharge program should be patient-centered, and focused on addressing the patient’s specific needs and preferences. In our program, we introduced the “follow-up nurse” instead of the “coach nurse” in Coleman’s model and “transitional care nurse” in Naylor’s model. The reason can be explained by establishing the hospital’s discharge-home care units, as one of the new policies in our health care system for chronic patients’ follow-up after hospital discharge (Behdasht.gov.ir). One of the major features of Coleman’s transitional care model is the important role of the nurse in improving the patient’s self-care and the family’s empowerment that we focused on it in our program. In Coleman model, the “coach nurse” abandons her/his traditional role of doing all the patient’s affairs and strengthens the “self-care” role of the patient through training (Coleman et al., 2006). Also, in discharge phase, an electronic patients’ record should be made on the electronic platform of the health care system to make the patient’s medical information accessible to all the health care team. 

After hospital discharge, cancer patient should be referred to the home health care centers for receiving transitional care when they need. The results of a study showed that many cancer patients, due to the inefficiency of the referral process in the health care system, are reluctant to be discharged from the hospital, and thus seek to benefit from health services for a longer time period (Walshe et al., 2008). If the patient needs home health care services, home care centers and their activities are introduced to the patient and family. Then, after signing the written consent and receiving an introduction letter from the hospital’s discharge-home care unit, patient with a summary of the medical record and assessment results by the follow-up nurse, is referred to the nearest home health care center. In Naylor’s model, a skilled and expert nurse, supports and helps the patient from discharge to home health care coordination (Naylor, 2004). 

In transitional care phase, it is necessary to perform triage to determine the level of patient’s care and to prioritize the time of the home visits. Therefore, a standard tool is needed to define the levels of necessary health services for cancer patients. Determining the level of care allows to access to a care plan tailored to the patient’s needs and to save resources (Wilner and Arnold, 2006). Studies showed that the triage coding system based on the patients’ needs is very important, in order to ensure a timely intervention in a large number of the patients (Dhiliwal et al., 2016; Hui and Bruera, 2017). After triage, it is necessary to develop a program, consisting of home visits and telephone follow-ups by the home health care team led by an experienced nurse for a period of four weeks. The program should be designed as a comprehensive patient-centered care program with the participation of the patient and family. In this program, all levels of health care services can be offered as a special package with different age groups and health conditions. They can be provided in 24 hours/7 days or intermittently for patients at home (Barasteh et al., 2020). In Naylor’s model the home health care nurse, has a supportive role and performs home visits to ensure the continuity of care after the discharge (Naylor, 2004). The results of a clinical trial with a 4-week home visit intervention using the Coleman’s model showed that the patient’s self-care role was activated, and also patient’s re-hospitalization rate has been significantly reduced (Coleman et al., 2006).

The current situation of the health care system shows that it is necessary to create a new electronic platform in the field of home health care services, in order to register patients’ electronic records at the time of hospital discharge, patients’ triage and telephone consultations. The telephone consultation system should be able to answer the questions of patients and their families in 24 hours/7 days by a skilled nurse with home health care experiences. In the new electronic platform, the patient’s records should also be accessible for planned home visits and referral to the higher levels of health care system. Peckham et al., (2019) showed that access to 24 hours/7 days support, facilitates the access to health care services and creates a sense of security for the family and the patient. In all the three models of patient care transition from hospital to home, telephone and in-person follow-ups by the nurse and the rest of the health care team are discussed) Naylor, 2004; Coleman et al., 2006; Hennessey and Suter, 2011 (During health care services by the home health care team, periodic assessments are also done according to the patients’ condition. Based on these assessments, the home health care program may be revised or based on the program’s objectives, the patient will be discharged from the home health care center. But then there would be a one-month telephone follow-up program. This cycle continues until the patient recovers or passes away (Shahsavari et al., 2018). In case the patient’s clinical condition deteriorates, he/she will be admitted and hospitalized in the nearest hospital to his/her place of residence (the referral process).

It seems that this integrated program can reduce unnecessary re-hospitalizations of cancer patients, also increase the patient’s self-efficacy and empower families. One of the advantages of our study, is its practical results in the future and its cost-effectiveness in the health care system. However, there are some limitations like the limited access to the expert and experienced participants in the field of home health care to conduct semi-structured interviews, as well as the development of executive protocols of the phase of health care service delivery by the home health care centers. These limitations may be the result of the evolving infrastructure of home health care centers in the community and their association with the private sector in our health care system. In order to investigate the effectiveness of the transitional cancer care program, pilot clinical trials and studies with a focus on the quality of life outcomes and cost-effectiveness, was recommended.

In conclusion, in the health care system of Iran, a program for care transition of cancer patients from hospital to home was developed, consisting of the three phases of “pre-discharge”, “post-discharge”, and “transitional care” with six protocols. The current situation of our health care system shows that in order to implement this comprehensive transitional cancer care program in this system, hospitals and home health care centers should work in the same direction. It is necessary to revise hospitals’ discharge program, and home health care center’s plan for admission and delivering health care services for cancer patients, and to establish an electronic platform which connects hospitals to home health care centers. Also, a pilot program is necessary to find the system advantages and weaknesses. Pilot clinical trials and prospective studies are essential by focusing on the QoL outcomes and cost-effectiveness.

## Author Contribution Statement

None.
